# A narrative review of the knowledge, attitudes, and practices of healthcare professionals toward diabetic retinopathy

**DOI:** 10.3389/fmed.2025.1536822

**Published:** 2025-08-07

**Authors:** Khisimusi Debree Maluleke, Saajida Mahomed

**Affiliations:** ^1^School of Medicine, College of Health Sciences, University of KwaZulu-Natal, Durban, South Africa; ^2^Department of Health, Limpopo Province, Sekororo District Hospital, Durban, South Africa

**Keywords:** knowledge, attitudes, practice, diabetic, retinopathy, complications, healthcare professionals

## Abstract

**Background:**

Diabetic retinopathy (DR) is a leading cause of preventable vision loss worldwide. Early screening and diagnosis are critical in mitigating vision loss in patients with diabetes. This review aims to analyze existing research on healthcare professionals’ (HCPs) knowledge, attitudes, and practices regarding DR screening.

**Methods:**

A literature search was performed using four electronic databases: Medline, Google Scholar, Science Direct, and EBSCOhost. The search terms included synonyms connected by the Boolean operator “OR.” This search covered quantitative, qualitative, and mixed-methods research studies. The appraisal was done using the Joanna Briggs Institute’s critical tool. A total of 59 published articles were analyzed.

**Results:**

Forty-seven articles (79.7%) assessed knowledge of HCPs, 30 (50.8%) assessed attitudes, and 42 (71.2%) assessed practices related to DR screening and referrals. The studies reveal significant differences in knowledge, attitudes, and practices regarding DR among various HCPs. There was variation in levels of knowledge amongst various categories of HCPs, with nine studies reporting 100% knowledge of DR. Positive attitudes ranged from 13 to 100%. Similar variations were reported regarding practices, with many HCPs not screening patients for DR. Inadequate training, lack of screening resources like an ophthalmoscope, dilating eye drops, and being inundated with other responsibilities were common reasons for the gaps in knowledge and practices.

**Conclusion:**

Non-ophthalmic HCPs’ knowledge, attitudes, and practices (KAP) regarding DR screening were unsatisfactory. The HCPs with an ophthalmic background had varying levels of KAP regarding DR, with some having good knowledge and others having deficiencies in applying screening guidelines and providing patient education. Regular in-service training is needed, particularly for non-ophthalmic HCPs, and resources should be available for screening at the primary care level.

## 1 Introduction

Diabetes mellitus (DM) is one of the leading non-communicable diseases that contributes to significant morbidity and mortality worldwide (1). The global prevalence of DM is 10.5% impacting around 536.6 million persons, and is expected to increase by 12.2% (783.2 million people) (1). As with many other diseases, the prevalence of DM differs from region to region. As per the International Diabetes Federation, countries in the Middle East, such as Kuwait, Bahrain, Qatar, and Egypt in the North Africa region, report the highest diabetic prevalence at 16.2% (2). The high prevalence of DM in this region is also influenced by a genetic predisposition to insulin resistance among various ethnic groups (3). In North America and the Caribbean region, the prevalence of DM was 14%, followed by Southeast Asia (10%), Western Pacific (9.9%), Europe (9.2%), and South and Central America (8.2%) (2). The lowest prevalence rate of DM is observed in the African region (4.5%), particularly in other countries of Eastern Sub-Saharan, comprising Uganda, Kenya, Malawi, Tanzania, Ethiopia, and Rwanda, with limited published data for some areas, such as in rural areas of Tanzania and Ethiopia, due to an inadequate surveillance system (4).

Diabetic retinopathy (DR) is a prevalent complication of diabetes (5). This is a complex diabetic microvascular complication initiated by chronic hyperglycemia, causing metabolic abnormalities in the retina, including neurodegeneration and inflammation (5). The progression of DR starts with retinal blood vessel damage, then thickening of the basement membrane, loss of pericytes due to apoptosis induced by hyperglycemia weakening capillary walls leaking to microaneurysm formation, and disruption of endothelial function causing fluid to leak from capillaries or blockage in the capillaries leading to cotton-wool spots due to the hypoxia (6). Diabetic retinopathy risk factors are linked to inadequate glycaemic management, diabetes duration, age, nephropathy, high blood pressure, high levels of lipids, obesity, pregnancy, previous eye surgery, and smoking (7).

There are two categories of diabetic retinopathy: non-proliferative and proliferative (5). Non-proliferative diabetic retinopathy (NPDR) is the initial progression stage of DR, and it is an asymptomatic stage characterized by the absence of neovascularization on the retinal sites (5). Proliferative DR (PDR) is the late stage of DR characterized by retinal neovascularization, usually with visual symptoms such as fluctuation of vision or reduced vision, seeing dark spots when looking in an open space due to hemorrhages in the vitreous space (8). Proliferative DR is considered a sight-threatening DR condition requiring urgent medical attention to prevent further vision loss or blindness (5). The healthcare professionals (HCPs) involved in the management of patients at risk for DR must understand the different stages and characteristics of DR so that appropriate interventions are offered promptly to prevent severe vision impairment or blindness. According to the epidemiological data from the global DR barometer, it has been observed that 28% of diabetic patients develop DR, while 42% develop diabetic macular edema (8). These findings emphasize the significance of early detection and treatment of eye issues related to diabetes (8).

In individuals between the ages of 20 and 70 in low- or middle-income countries, DR is the primary reason for blindness or moderate-to-severe vision impairments (1). Nearly 80% of adults, equating to 4.2 million adults, and 655,000 adults have some form of DR, which is more than twice in Mexican Americans and almost three times as common in African Americans (1). Globally, DR had a prevalence of 22.3% according to a 2021 systematic review (9). The prevalence of sight-threatening DR and clinically significant macular edema is 6.2% and 4.1%, respectively (9). Africa and North America have the largest prevalence of DR at 35.9% because of the growing diabetic population, while South and Central America have the smallest prevalence at 13.4% (9). According to a 2021 systematic review, the prevalence of DR in the Sub-Saharan African (SSA) region varies from 13 to 82%, while the sight-threatening DR ranges from 2.1% to 51.4% based on a systematic review reported in 2021 (10).

It is the responsibility of the HCPs managing a patient with diabetes to screen or refer the patient for screening for DR. Healthcare professionals’ knowledge, attitudes, and practices (KAP) of DR screening and referrals play a vital role in preventing vision impairment in people with diabetes. Late screening of patients with diabetes due to poor referral systems can lead to permanent vision impairment or blindness (7). While problems within the healthcare system may lead to delays in the diagnoses and management of patients, a lack of awareness among HCPs regarding the significance of DR screening can also be an important contributory factor (11). Alarmingly, over 37% of diabetic patients globally suffer from DR because of delays in referring them for an eye screening (8).

After critically analyzing the literature discussed, screening for DR appears to be sub-optimal, and there is therefore a need to document the gaps in the existing practices of HCPs regarding DR screening. Whilst previous reviews have looked at the general complications of DM (12), this is the first narrative review to focus specifically on DR. We aimed to evaluate and summarize the key findings of published studies that have investigated knowledge, attitudes, and practices of HCPs regarding DR. This review will offer important insights and strategies to strengthen DR screening. Additional advantages include supporting ongoing training to improve healthcare providers’ comprehension of DR screening and referral procedures. This is especially important for non-ophthalmic providers.

## 2 Method and materials

### 2.1 Literature search strategies and eligibility

Before commencing the literature search, the strategy and eligibility for inclusion and exclusion criteria for a review were developed. A systematic search was done to identify published articles on the KAPs of HCPs about DR. Five electronic databases were searched, including Medline (via the PubMed and Ovid interfaces), Google Scholar, Scopus, Science Direct, and EBSCOhost. We used the “building blocks” approach, often used in reviews, to create thorough search strategies. We organized search terms into categories representing different HCPs involved in treating patients with diabetes (including those impacted by DR). We also broadened the search terms by including synonyms and using the Boolean operators to connect them. The keywords include “knowledge OR attitude OR practice, diabetic retinopathy OR diabetic complications, healthcare professionals OR workers OR providers OR physicians OR nurses OR doctors OR general practitioners OR optometrists OR ophthalmologists.” The search was restricted to articles from earlier research studies from 1996 to 2023. Table 1 presents the components of the criteria for inclusion and exclusion.

**Table 1:** Criteria for inclusion and exclusion.

Inclusion criteria	Exclusion criteria
Articles reporting on original research	Duplicates
Published in peer-reviewed journals	Gray literature such as technical reports, news reports, blogs, policies, and web-based guidelines
Articles published in the English language	Letters to the editors
The study population must be healthcare professionals (HCPs)	Book reviews and chapters
Academic reports such as theses or dissertations from Institutional Repositories	Opinion pieces and commentaries
Studies conducted in public and private settings	The study population included students, patients, or the general population

### 2.2 Article selection

Following an in-depth search, all retrieved articles were entered into Mendeley 2.110.0 software (2024 Elsevier, Mendeley Ltd., London). The identified duplicates were removed using the duplicates command. Relevant articles were selected in three phases. In phases 1 and 2, the titles and abstracts of articles were screened by the first author (KDM) with the help of two colleagues working in the same organization as KDM, and irrelevant articles were excluded. In phase 3, the full-text manuscripts were carefully assessed. The articles of studies that met pre-defined inclusion criteria in Table 1 were selected. KDM decided to include relevant studies, but the disagreements were discussed to reach a consensus.

### 2.3 Data extraction and quality assessment

KDM carefully assessed the title and abstract of each study, and data related to the topic were extracted. The quality assessment was done using two Joanna Briggs Institute’s (JBI) critical appraisal tools for methodological appropriateness, including analytic methods, with one revised version containing 8 items for analytical cross-sectional studies, and another containing 10 items for qualitative studies (13). In this review, the level of quality was assessed based on the elements of methodological appropriateness from the JBI critical appraisal tool, with the results included in Supplementary Tables 1 and 2. The quality assessment scores employed for a review include methodologically strong (with <2 missing criteria), moderate (with 2–3 missing criteria), and weak (with >2) (14).

## 3 Results

### 3.1 Identification of studies

**Figure 1:**
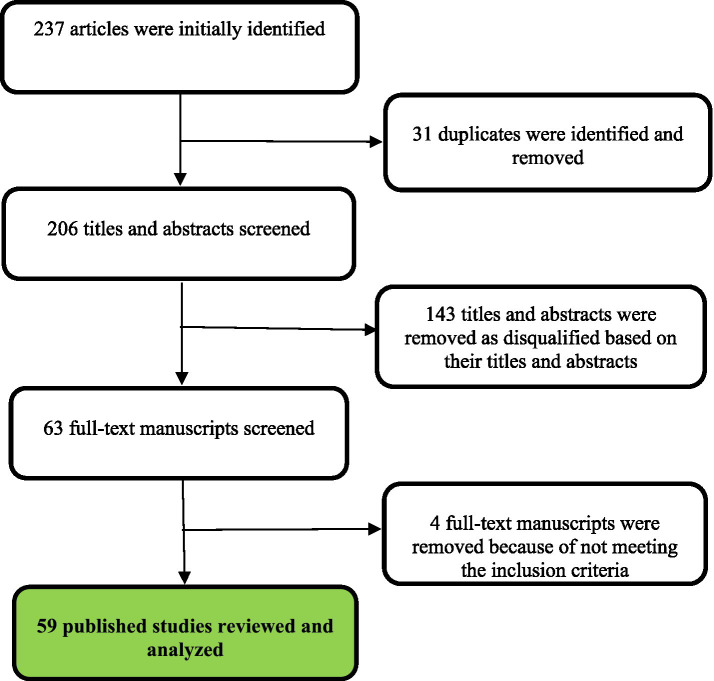
Literature selection.

The initial search identified a total of 237 articles. Following the exclusion of 31 duplicates, the titles, and abstracts of 206 articles of published studies underwent a screening process to identify published articles relevant to this review. Subsequently, 123 articles were excluded based on the pre-defined inclusion criteria (Table 1). In addition, 24 articles were excluded after reviewing the full-text manuscripts as the focus was on the treatment of DR. Finally, 59 published studies were included and analyzed in this review. A summary of the literature search and selection stages has been provided in Figure 1.

### 3.2 Summary of included studies

**Figure 2:**
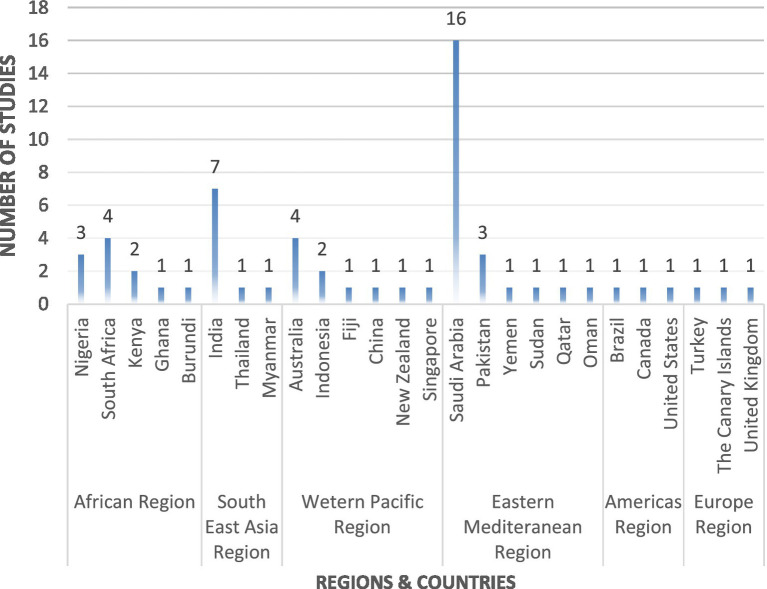
The number of studies included per region and their respective countries.

The studies included originated from all six World Health Organization (WHO) regions, with the majority (*n* = 23) being from the Eastern Mediterranean region, followed by 11 from the African region and 10 from the Western Pacific region (Figure 2). There were only three studies that were conducted in the Americas and European regions. In terms of individual countries, 16 studies were conducted in Saudi Arabia, followed by seven in India (15–73). The study settings included public and private healthcare sectors, and the study populations included various categories of HCPs who manage diabetes and DR. The sample sizes ranged from eight HCPs to 710 physicians.

### 3.3 Study quality

The reviewed studies in this analysis were characterized by clearly defined objectives and the use of the appropriately selected methodologies, as determined through evaluation with the Joanna Briggs Institute’s (JBI) critical appraisal tool (13, 74–76). Thirty-six quantitative studies demonstrated a moderate JBI quality level (16–26, 28–32, 34, 35, 37–41, 43–47, 49, 52–54, 58, 60, 62, 63, 65, 66, 68, 70, 71). The other 18 showed a strong level in Supplementary Table 1 (15, 27, 29, 30, 33, 36, 42, 44, 48, 51, 55, 57, 59, 61, 64, 65, 69, 72). Three qualitative studies demonstrated moderate levels of quality assessment (56, 67, 73), and one showed a strong JBI quality assessment (Supplementary Table 2) (50).

### 3.4 Categories of healthcare professionals

The HCPs included in this review are primary healthcare nurses, primary care physicians (general practitioners or family physicians, and internists), ophthalmic care professionals (ophthalmologists, optometrists, and ophthalmic nurses), medical residents, diabetologists, dietitians, laboratory scientists, physical therapists, general nursing personnel (professional registered nurses, and staff nurses), paramedical personnel, and other clinical officers involved in the management of diabetes (Table 2). Some of the included studies used the terms “medical practitioner,” “medical officer,” and “physicians” interchangeably when referring to the doctors (17, 19, 20, 22, 24, 25, 28, 29, 32, 40, 41, 50, 52, 53, 61, 66, 69, 73). This review presents all HCPs as described in their respective articles.

**Table 2:** Summary characteristics of reviewed studies.

Studies	Countries (sites)	Sample	Design, methods, and tools	Assessed knowledge, attitudes, and practice (KAP) elements	Main findings
Abdool et al. 2016 (15)	South Africa (eThekwini)	104 HCPs (42 primary healthcare (PHC) nurses, 5 ophthalmic nurses, 30 medical officers, 23 optometrists, 17 ophthalmologists, and 9 managers)	Cross-sectional study, paper-based survey, and self-administered structured questionnaire with “yes/no/not sure” responses	Practice	About 40.6% of medical officers (MOs) performed fundoscopy with 71.9% reported knowing how to perform direct ophthalmoscopy, 43% of PHC nurses only take a case history and refer those with eye problems, 80% of ophthalmic nurses take a case history, dilate patients for fundoscopy including screening of cataracts and glaucoma, 40% of optometrists discussed ocular complication of diabetes, 72% performed direct ophthalmoscopy, and 82.4% of ophthalmologists used fundus cameras to detect DR
Abdool et al. 2020 (16)	South Africa (Waterburg & Capricorn Districts)	14 PHC nurses, 17 ophthalmologists, 23 optometrists, 10 ophthalmic nurses	Cross-sectional study, paper-based survey, and self-administered structured questionnaire with “yes/no/not sure” responses	Practice	All PHC nurses had no knowledge of DR screening procedures except taking a case history and vital sign measurements and then referring those presenting with visual problems to eye clinics, 94.7% of ophthalmologists showed high knowledge in fundoscopy and DR classification, followed by 91.3% of optometrists, and 10% of the ophthalmic nurses knew how to perform fundoscopy.
Abdulsalam et al. 2018 (17)	Nigeria (North-western Nigeria)	105 Physicians (61 general practitioners (GPs), 37 residents/ senior MOs, and 7 other consultants, principals, and chief MOs)	Cross-sectional study, paper-based survey, and self-administered structured questionnaire with “yes/no/not sure” for knowledge and 5-point Likert scale responses for attitudes, and practices.	KAP	Approximately 63.8% of physicians were aware of the most effective techniques for delaying the onset and progression of DR, 71.5% agreed that the lack of an ophthalmoscope is the main barrier to eye screening, and 36.2% performed a routine eye examination.
Abu-Amara et al. 2019 (18)	Saudi Arabia (Riyadh)	355 non-ophthalmic professionals (22 consultant specialists, 20 senior specialists, 119 specialists, 23 residents, and 171 GPs)	Cross-sectional study, paper-based survey, and self-administered structured questionnaire with a 5-point Likert scale for attitudes, and “yes/no/not sure” responses for knowledge and practices	KAP	More than half (54.3%) of non-ophthalmic professionals knew that diabetes could damage the eye, and 68.7% indicated that lack of resources, training, and being busy with other health issues were barriers. Only 31.3% had positive attitudes, and 40.8% had good practices by referring all diabetic patients for eye screening.
Ahmed et al. 2020 (19)	Saudi Arabia (Dammam, Jeddah, and Riyadh)	709 Physicians (294 family medicine, 277 GPs, and 138 others)	Cross-sectional study, paper-based survey, and self-administered structured questionnaire with “yes/no/not sure” responses	Knowledge and practice	About 38.9% of physicians knew that complicated diabetes could damage the eye, and 19.7% showed good practices by referring patients with diabetes to an ophthalmologist for an eye examination immediately after diagnosis.
Al-Rasheed and Al-Adel 2017 (20)	Saudi Arabia (Riyadh)	216 primary care physicians (PCPs), i.e., 142 family medicine, 10 pediatricians, 8 internal medicine, and 56 general physicians	Cross-sectional study, paper-based survey, and self-administered structured questionnaire with “yes/no/not sure” responses	Knowledge and practice	Only 19% of PCPs were aware of anti-vascular endothelial growth factors caused by DR, 65% routinely refer diabetic patients to ophthalmologists, and 24% correctly refer patients with type 1 diabetes.
Alanazi et al. 2018 (21)	Saudi Arabia (Tabuk City)	87 GPs	Cross-sectional study, paper-based survey, and self-administered structured questionnaire with “yes/no” for both knowledge and practice, and “agree/disagree/I do not know” responses for attitudes	KAP	Only 24.1% of GPs could identify pregnancy as the risk factor for having DR, 43.7% knew how to detect retinal detachment, and 28.7% could detect vitreous hemorrhage. But 43.7 and 28.7% knew retinal detachment. Most GPs (90.8%) showed positive attitudes after disagreeing that an eye examination for diabetic patients is only indicated once vision is affected, and 87.4% had good practices by referring patients yearly.
Alasqah et al. 2020 (22)	Saudi Arabia (Qassim)	106 PCPs (51 family and 5 internal medicine, and 5 pediatricians, 36 GPs, and 3 others)	Cross-sectional study, paper-based survey, and self-administered structured questionnaire with “agree/disagree” responses	KAP	The majority (88%) of physicians knew that DR is a common cause of vision impairment, 95% agreed that patients should be asked about vision during every visit, 87% agreed to have an ophthalmoscope in their clinics, 38% performed ophthalmoscopy, and 90% of PCPs had positive attitudes toward DR screening after agreeing that they should be actively involved in fundoscopy.
Al-Ghamdi et al. 2017 (23)	Saudi Arabia (Taif)	180 GPs	Cross-sectional study, paper-based survey, and self-administered structured questionnaire with “yes/no/I do not know” and 5-point Likert scale responses	KAP	Almost all (97.2%) GPs knew the prevalence of diabetes and DR, 92.8% had positive attitudes toward DR by agreeing that early detection and treatment could prevent vision loss, and 43.9% had good practices of DR in performing funduscopic examination.
Alhejji et al. 2020 (24)	Saudi Arabia (Al-Hasa)	141 PCPs (56 family medicine, 10 internal medicine, and 75 GPs)	Cross-sectional study, paper-based survey, and self-administered structured questionnaire with “yes/no” responses	KAP	Over half (56%) of PCPs had good knowledge, 36.9% educated patients about the early detection of diabetic complications, and 24.1% correctly referred according to the *American Academy of Ophthalmology Guidelines*.
Almoitairy et al. 2021 (25)	Saudi Arabia (Riyadh)	371 Physicians (59 internal and 56 family medicine, 54 gynecologists, 25 emergency medicine, 20 ENTs (ear, nose, and throat specialists), 19 pediatricians, general surgeons, 15 urologists, 15 anesthesia, 14 radiologists, 12 orthopedics, 12 dermatologists, 11 GPs, 10 cardiac surgeons, 9 neurologists, 8 community medicine, 6 physical medicine, and 8 others)	Cross-sectional study, paper-based survey, and self-administered structured questionnaire with “yes/no/I do not know” responses for both knowledge and attitude.	Knowledge and attitude	Only 19.1% of physicians had high knowledge of DR, and 59.6% had positive attitudes by believing that performing ophthalmoscopy could assist in early detection
Al-Rashidi et al. 2020 (26)	Saudi Arabia (Qassim)	96 GPs	Cross-sectional study, paper-based survey, and self-administered structured questionnaire with “yes/no/I do not know” responses	Knowledge and practice	Just 26.4% of GPs showed good knowledge about DR by referring type 1 diabetic patients according to the *American Academy of Ophthalmology Guidelines*, whereas 74% referred all type 2 diabetic patients to ophthalmologists.
Alsaedi et al. 2022 (27)	Saudi Arabia (Western Region)	351 HCPs (135 residents, 112 nurses, 29 consultants, 29 specialists, 26 pharmacists, and 20 optometrists)	Cross-sectional study, paper-based survey, and self-administered structured questionnaire with “yes/no/I do not know” responses	Knowledge	Only 3.7% of HCPs knew how to diagnose DR
Alzaidi et al. 2016 (28)	Saudi Arabia (Taif)	101 Physicians (44 internal medicine, 25 general surgeons, 17 family medicine, 12 ophthalmologists, and 3 other specialists)	Mixed methods study, paper-based survey, and self-administered semi-structured questionnaires with a 5-point Likert scale and open-ended responses	KAP	Over 70% of physicians had good knowledge of DR, 94% believed all patients with diabetes should undergo a periodic eye examination, and 90% could detect and prevent DR correctly
Anwar et al. 2019 (29)	Pakistan (Islamabad, and Rawal-Pindi)	36 PCPs (27 GPs, 2 family physicians, and 7 internists)	Cross-sectional study, paper-based survey, and self-administered structured questionnaire with multiple-choice responses	Knowledge and practice	Mean scores of good knowledge that diabetes could damage the eye among GPs, family physicians, and Internists were 41.7, 42, and 46.6%, respectively. Only 5% of PCPs performed an ophthalmoscopy regularly
Babelgaith et al. 2013 (30)	Yemen (Mukalla)	73 HCPs (37 doctors, 19 pharmacists, and 17 nurses)	Cross-sectional study, paper-based survey, and self-administered structured questionnaire with 5-point Likert scale responses	Attitude	All (100%) HCPs in the study have expressed positive attitudes toward diabetes and its complications, such as DR
Babu et al. 2021 (31)	India (Tertiary Institution)	108 non-ophthalmic specialists (8 professors/ HODs, 41 associate professors, 36 senior residents, and 33 junior residents)	Cross-sectional study, paper-based survey, and self-administered structured questionnaire with “yes/no” for knowledge, practices, and “agree/disagree” responses for attitudes	KAP	Over three-quarters (75.6%) of participants had excellent knowledge that DR has a damaging effect on the eye, over 87.6% with positive attitudes believed that DR screening is critical in preventing eye damage due to complicated diabetes, and only 45.5% had good practice of DR by referring to ophthalmologists for an eye examination.
Bogunjoko 2015 (32)	Nigeria (Ogun State)	16 Medical officers	Cross-sectional study, telephonic survey, and self-administered structured questionnaires with “yes/no/I do not know” responses	KAP	All (100%) medical officers knew that diabetes could affect the eye, 43% believed that patients with diabetes should have monthly eye check-ups, and all (100%) referred diabetic patients to ophthalmologists for an eye examination.
Barakat et al. 2023 (33)	Saudi Arabia	267 Ophthalmologists and 42 others	Cross-sectional study, paper-based survey, and self-administered structured questionnaire with “yes/no” responses	Practice	More than half (54%) of all respondents had good practice in managing patients with DR.
Carlos et al. 2007 (34)	Brazil (São Paulo)	168 endocrinologists	Cross-sectional study, paper-based survey, and self-administered structured questionnaire with “yes/no” responses	Practice	Only 36.9% of endocrinologists referred patients with type 1 diabetes, and 86.9% referred patients with type 2 diabetes to ophthalmologists for an eye examination.
Chelliah et al. 2020 (35)	India (Tami Nadu)	103 Non-ophthalmic doctors	Cross-sectional study, paper-based survey, and self-administered structured questionnaire with “yes/no” responses	Knowledge	Over a third (35%) of non-ophthalmic doctors knew the correct schedule for DR screening. From this 97% knew that diabetes could affect the eye,18.4% referred all patients with diabetes to ophthalmologists for an eye examination,
Daly 2014 (36)	New Zealand (Auckland)	287 Nurses (210 practice nurses, 49 district nurses, and 28 specialist nurses)	Cross-sectional study, telephonic survey, and self-administered structured questionnaires with “yes/no” responses	Knowledge	Most (86%) nurses knew how to identify diabetes-related complications like DR.
Delorme 1998 (37)	Canada (Quѐbec and Chaudiѐre Appalaches)	645 GPs and 96 residents	Cross-sectional study, paper-based survey, and self-administered structured questionnaire with “yes/no/I do not know” responses	Knowledge and attitude	Just 13% of GPs and 60% of residents knew that the initial eye screening for DR should be done after the onset of diabetes, and 70% had negative attitudes when they felt not competent to screen diabetic patients for DR.
Dickson et al. 1996 (38)	Australia (Victoria)	500 GPs	Cross-sectional study, self-administered structured questionnaires with a paper-based survey, “yes/no” responses	Practice	The majority (88%) of GPs often refer diabetic patients to ophthalmologists for an eye examination.
Edwiza et al. 2021 (39)	Indonesia (Bandung)	115 GPs	Cross-sectional study, paper-based survey, and self-administered structured questionnaire with “yes/no” responses for knowledge and practice, and a 5-point Likert scale for attitudes	KAP	Most (85.2%) GPs had good knowledge that diabetes could cause eye damage due to DR, 100% had positive attitudes believed in DR screening, and 32% demonstrated good practices of DR by referring patients with diabetes for an eye examination
Elnagieb and Saleem 2017 (40)	Sudan (Khartoum)	225 Medical doctors (100 GPs, and 125 medical residents)	Cross-sectional study, paper-based survey, and self-administered structured questionnaire with “yes/no/I do not know” responses	KAP	The majority (90.8%) of medical doctors knew that the retina could be affected by diabetes, 51% agreed to do eye examinations for all diabetic patients, 42% did visual acuity tests, and 30% did fundoscopy.
Erdem 2020 (41)	Turkey	92 PCPs	Cross-sectional study, online-based survey (via WhatsApp messages), and self-administered structured questionnaire with “yes/no” responses	Knowledge and practice	Almost all (97.8%) of PCPs had a Snellen chart, 98.9% had a direct ophthalmoscope, only 23.9% referred patients to ophthalmologists at the time of diagnosis, 20.7% referred patients a year after first diagnoses, 10.8% referred patients at the 2-year interval, 4.3% refer at 6-month intervals, and 3.3% refer over 2-year intervals.
Fernández-Gutliѐrrez et al. 2023 (42)	The Canary Islands (Tenerife)	165 PHC nurses	Cross-sectional study, paper-based survey, and self-administered structured questionnaire with “matching/lesion observed/suspected lesion” responses	Knowledge	More than two-thirds (68.4%) of PHC nurses knew the difference between normal and diabetes-affected retinal images
Fatima and Ahmad 2018 (43)	Pakistan	95 GP	Cross-sectional study, paper-based survey, and self-administered structured questionnaire with “yes/no” responses	KAP	Most (90.5%) GPs agreed that diabetic patients need eye examination once a year, 78% with a positive attitude believed optometric services could assist in DR screening, and 64.2% referred diabetic patients to optometrists.
Foster 1996 (44)	United States (New York State)	23 Optometrists	Cross-sectional study, mail-based survey, and self-administered structured questionnaire with “yes/no” responses	Practice	Over a third (38.5%) of optometrists who graduated before 1964 performed dilated fundoscopy compared to 47% of those who graduated between 1964 and 1983, and all (100%) optometrists who graduated after 1984 performed dilated fundoscopy.
George et al. 2019 (45)	Singapore	230 Optometrists	Cross-sectional study, mail survey, and self-administered structured questionnaire with “yes/no” and open-ended responses	Attitude	Nearly three-quarters (71%) of optometrists felt they should undertake regular continuous professional education to improve their primary eye care, including diagnosing eye diseases like DR.
Gharsangi et al. 2021 (46)	India (Himachal Pradesh)	102 Nurses	Cross-sectional study, paper-based survey, and self-administered structured questionnaire with “correct/ incorrect” responses	Knowledge	Most (88.2%) nurses knew that patients with diabetes are susceptible to microvascular complications of diabetes, like DR and others.
Ghosh 2007 (47)	India (South 24 Parganas, and West Bengal)	36 Optometrists, and 242 GPs	Cross-sectional study, paper-based survey, and self-administered structured questionnaire with “correct/ incorrect” responses	Knowledge	Just 21.9% of GPs knew the magnitude of DR, 26% knew the risk factors of DR, and 32.2% knew the management of DR. For optometrists, 13.9% knew the magnitude of DR, 22.2% knew the risk factors of DR, and 16.7% knew management of DR.
Goodman et al. 1997 (48)	South Africa (Cape Town)	35 HCPs (12 doctors, 10 PHC nurses, 7 registered nurses and 6 staff nurses)	Cross-sectional study, paper-based survey, and self-administered structured questionnaire with “yes/no” responses	Knowledge	All (100%) HCPs had a good knowledge of chronic complications of diabetes such as DR and others.
El-Hajj et al. 2018 (49)	Qatar	126 Pharmacists	Cross-sectional study, online, and paper-based survey with a self-administered structured questionnaire with 5-point Likert scale responses	Practice	Half (50%) of pharmacists had good practices of diabetes and its chronic complications, like DR, by giving patients regular health education.
Hipwell et al. 2014 (50)	United Kingdom (in 3 screening programs)	8 PCPs	Descriptive qualitative study, paper-based survey, and self-administered semi-structured questionnaire with “yes/no” and open-ended responses	Knowledge	The primary care physicians had clear overall knowledge of DR, including the screening program for DR.
Jagun et al. 2020 (51)	Nigeria (Ogun State)	154 HCPs (78 doctors, 51 nurses, and 25 others)	Cross-sectional study, paper-based survey, and self-administered structured questionnaire with “yes/no/not sure” responses	Knowledge	Over two-thirds (70%) of HCPs were aware that diabetes could lead to DR and other microvascular complications.
Khan et al. 2011 (52)	Saudi Arabia (Al-Hasa region)	99 Primary care physicians	Cross-sectional study, paper-based survey, and self-administered structured questionnaire with correct/ incorrect responses	KAP	Most (85.9%) PCPs knew the prevention of microvascular complications like DR is important, 13.2% had positive attitudes by believing in the prevention of DR, and 20.6% referred patients with diabetes.
Khandekar et al. 2008 (53)	Oman (North Sharqiya)	36 Physicians (14 family physicians, 9 hospital physicians, 1 diabetologist, and 12 other types of doctors)	Cross-sectional study, paper-based survey, and self-administered structured questionnaire with “yes/no” responses	KAP	Only 58% of physicians knew eye parts and had knowledge of fundoscopy, 74% with positive attitudes believed in fundoscopy for DR screening, and 50% had an opportunity to perform ophthalmoscopy due to the availability of this medical instrument.
Kiely et al. 2017 (54)	Australia	587 Optometrists	Cross-sectional study, mail survey, and self-administered structured questionnaire with “very confident/not confident/ never learn to perform” responses	Practice	About 93% of optometrists had high confidence in practicing good optometry, including diagnosing diseases like DR.
Kumar et al. 2020 (55)	India (Tamil Nadu)	8 HCPs (5 ophthalmologists, 2 diabetologists, and 1 dietician)	A descriptive qualitative study, a paper-based survey, and a semi-structured questionnaire.	Knowledge	Four themes were recognized: living with diabetes, care-seeking practices, and awareness of DR, including barriers to DR screening. The overall results showed that HCPs have good knowledge of DR.
Kumar et al. 2023 (56)	Thailand	9 nurses, 8 nursing aides or assistants, a nutritionist, 2 ophthalmologists, 2 pharmacists, a physical therapist, a registered nurse, a registrar, a technician, and a laboratory scientist	A descriptive qualitative, focus group discussion (FGD), and an unstructured questionnaire	Knowledge	Different themes showed an overall knowledge of diabetes, self-care behaviors on diabetes, awareness of DR, barriers to DR screening, and suggested solutions to address identified barriers.
Kupitz et al. 2014 (57)	Kenya (Kenyatta National Hospital)	46 HCPs (25 physicians, 14 nurses, 6 clinical officers, and 1 nutritionist)	Cross-sectional study, paper-based survey, and self-administered structured questionnaire with 5-point Likert scale responses	KAP	The majority (91%) of participants saw diabetes and DR as urgent health problems, 52% with positive attitudes toward DR believed in efficient detection and referrals, and 30% saw improved outreach services as the most pressing area of need.
Lestar et al. 2023 (58)	Indonesia (Jakarta)	92 GPs	Cross-sectional study, online-based survey, and self-administered structured questionnaire with “yes/no” responses	KAP	Nearly 90% of GPs had good knowledge of DR detection, all (100%) GPs had positive attitudes toward DR screening to prevent vision loss, and 4.3% showed good practices by performing visual acuity testing and ophthalmoscopy in diabetic patients.
Malik et al. 2023 (59)	Pakistan (Karachi)	40 non-ophthalmic Surgeons	Cross-sectional study, online-based survey (via Google Forms), and self-administered structured questionnaire with “yes/no” responses	Knowledge and practice	Approximately 77.5% of the surgeons demonstrated excellent knowledge of DR, and 75% had good referral practices to ophthalmologists for DR.
McCarty et al. 2000 (60)	Australia	577 Ophthalmologists	Cross-sectional study, mail-based survey, and self-administered structured questionnaire with “Almost never/sometimes/often/ almost always/refer elsewhere” responses	Knowledge	Only 19% of ophthalmologists specializing in vitreoretinal surgery showed extensive knowledge in managing DR.
Menash 2013 (61)	Ghana (Regional Hospitals)	91 Medical Practitioners	Cross-sectional study, paper-based survey, and self-administered semi-structured questionnaire with “yes/no” responses	KAP	About 96% of medical practitioners had good knowledge that complicated diabetes could damage the eye, 92% had positive attitudes toward DR screening, and only 34% had good practices of DR by referring diabetic patients to ophthalmologists for eye examination.
Muecke 2008 (62)	Myanmar (Yangon)	100 GPs	Cross-sectional study, paper-based survey, and self-administered structured questionnaire with “yes/no” responses	Knowledge and practice	Almost all (99%) GPs were aware that complicated diabetes could lead to vision loss or blindness, and 49% never performed fundoscopy on diabetic patients.
Namperumalsamy et al. 2004 (63)	India (Southern India)	200 paramedical personnel	Cross-sectional study, paper-based survey, and self-administered structured questionnaire with “yes/no” responses	KAP	Mere 2.5% of paramedical personnel knew that diabetes could damage the eye, 81% with positive attitudes believed that screening for DR could prevent blindness, and 56.5% had good practice of DR by providing educational materials.
Niyonsavye 2015 (64)	Burundi (at the District and Regional hospitals)	81 GPs	Cross-sectional study, paper-based survey, and semi-structured questionnaire with “yes/no” and open-ended responses	KAP	Just 24.7% of GPs knew the risk factors of DR, 92.6% with positive attitudes believed in the importance of DR screening program, and 22.2% were testing vision for patients with diabetes.
Oenga 2012 (65)	Kenya (at the Provincial Hospitals)	91 GPs	Cross-sectional study, paper-based survey, and self-administered structured questionnaire with “yes/no” responses for knowledge and practices, and a 5-point Likert scale for attitudes	KAP	All GPs knew that the eye and visual function could be impaired by complicated diabetes, 87.9% had positive attitudes toward DR, and 38.5% referred patients with diabetes to ophthalmologists for an eye examination.
Pradhan et al. 2018 (66)	Saudi Arabia (Kathmandu)	45 Physicians (20 government physicians, 21 from private hospitals, 3 from NGO-run hospitals, and 1 from a community-based hospital)	Cross-sectional study, paper-based survey, and self-administered structured questionnaire with “yes/no/I do not know” responses	KAP	All physicians were aware that diabetes could damage the eye, 98% disagreed that eye screening for patients with diabetes is required once vision gets affected, and 56% agreed that they routinely do direct ophthalmoscopy.
Ram et al. 2022 (67)	Fiji	14 Community Health Workers (CHWs)	A descriptive qualitative study, using FGD, and an unstructured questionnaire	Knowledge and practice	Before the training on DR, CHWs lacked information on DR, including referral practices. After the training, all CHWs demonstrated improved knowledge of DR.
Raman et al. 2006 (68)	India (South India)	159 GPs	Cross-sectional study, telephonic-based survey, and self-administered structured questionnaire with “yes/no” responses	KAP	Almost a third (31.3%) of GPs felt that all patients with diabetes should undergo an eye examination every 6 months, 53.3% felt patients should be examined yearly, 15.4% felt that patients should be examined biannually, and 1.3% performed ophthalmoscopy.
Shah et al. 2017 (69)	Pakistan	56 doctors, 29 optometrists, and 11 orthoptists	Cross-sectional study, paper-based survey, and self-administered semi-structured questionnaire with “yes/no” responses	Attitude	Most participants in each category (75% of ophthalmologists, 86% of optometrists, and 90.9% of orthoptists) believed that sharing tasks would not degrade the level of care. They also suggested implementing standardized training for all eye care workers to share tasks.
Thirunavukkarasu et al. 2021 (70)	Saudi Arabia (Alijouf, Hail, the Northern Border, and Tabuk)	274 PCPs (164 residents, 77 specialists, and 33 consultants)	Cross-sectional study, paper-based survey, and self-administered structured questionnaire with 5-point Likert scale responses	KAP	Among all categories of PCPs, 21.5% demonstrated a good knowledge of DR, 15% displayed positive attitudes, and 29.2% exhibited good practices related to the management of DR
Wright et al. 2001 (71)	Australia (Optometric Association)	368 Optometrists	Cross-sectional study, paper-based survey, and self-administered structured questionnaire with 5-point Likert scale responses	Attitude and practice	Over half (57%) of optometrists read the “Clinical Practice Guidelines for Diabetic Retinopathy.” Sixty-five percent reported referring to the Retinopathy Chart. Seventeen percent with negative attitudes agreed that the guidelines were not practical or feasible, showing a negative attitude.
Xulu-Kasaba et al. 2021 (72)	South Africa (Kwa-Zulu-Natal)	77 HCPs in eye health services (3 ophthalmologists, 2 ophthalmic MOs, 38 optometrists, and 24 ophthalmic nurses and 24 clinical managers in ophthalmic care)	Cross-sectional study, paper-based survey, and self-administered structured questionnaire with 5-point Likert scale responses	KAP	Most (81.6%) participants had good knowledge of screening diabetic patients for DR and assisted ophthalmologists in theater activities. 69% showed a positive attitude toward DR screening, and 73.3% had good practice in DR by following the appropriate protocols.
Yan 2012 (73)	China	22 Physicians (8 ophthalmologists, 9 ENTs, and 5 internists), and 22 Village Health Workers	Descriptive qualitative study, FGD, and a researcher-administered unstructured questionnaire	Knowledge and attitudes	All physicians, including ophthalmologists, ENTs, internists, and village health workers, demonstrated a good understanding of the prevalence, severity, diagnosis, and treatment of DR. However, the physicians with positive attitudes believed that cost was the primary barrier to patients’ compliance with diabetic care and continuous treatments to avoid microvascular complications of diabetes, like DR.

### 3.5 Methods of assessing healthcare professionals’ knowledge, attitudes, and practices

Fifty-three of the 59 studies used a cross-sectional design (15–49, 51–54, 57–66, 68–72, 77–87), five were descriptive qualitative studies (50, 55, 56, 67, 73), and one study employed a mixed-methods approach (28). Fifty-one studies used self-administered structured interview questionnaires with closed-ended questions (15–27, 29, 30, 32–42, 44–46, 49, 51–60, 62–68, 70–72, 77–79, 81–88). Five studies used semi-structured questionnaires with closed and open-ended questions (28, 50, 55, 61, 69), and three studies used unstructured questionnaires (56, 67, 73). The studies employed various methods to gather data, such as paper-based, telephone, and online surveys, and focus group discussions (FGDs) for qualitative research (15–73, 85). Twenty-three studies assessed knowledge, attitudes, and practices (17, 18, 21–24, 28, 31, 32, 39, 40, 43, 53, 57, 58, 61, 63–66, 69, 70, 72, 85), three assessed knowledge and attitudes (25, 37, 52), five assessed knowledge, and practices (20, 26, 29, 59, 62), and one study assessed attitudes and practices toward DR (71). Fourteen studies assessed knowledge only, 10 of which used structured questionnaires comprising “yes/no/I do not know” responses (27, 35, 36, 41, 42, 46–48, 50, 51), one used a questionnaire with “almost never/sometimes/often/almost always/refer somewhere” responses (60), and four were focus group discussions (55, 56, 67, 73). Two studies assessed attitudes only with one study using a structured questionnaire with a 5-point Likert scale (1 = strongly agree, 2 = agree, 3 = neutral, 4 = disagree, 5 = strongly disagree) (30) and the other used a questionnaire with “yes/no/I do not know/not sure” responses (45). Nine studies assessed practices only with seven using questionnaires with “yes/no/I do not know” responses (15, 16, 33, 34, 38, 44, 68), one using a questionnaire with “very confident/not confident/never learn to perform” responses (54), and another one used a questionnaire with 5-point Likert scale responses (49).

### 3.6 Healthcare professionals’ knowledge of diabetic retinopathy

Of the 59 studies, 47 (79.7%) assessed the knowledge of DR among different categories of HCPs. Most studies (over 90%) utilized a common set of DR knowledge-related questions (17–29, 31, 32, 35–37, 39–43, 46–48, 50–53, 55–67, 69, 70, 72, 73, 85). These questions encompassed various aspects of diabetes and its impact on the retina. Topics included the knowledge of DR prevalence among individuals affected by diabetes, the effects caused by complicated diabetes on vision, eye complications linked to diabetes, the preferred method for evaluating DR in different resource settings, progressive stages of DR with their respective clinical manifestations, and the risk factors were linked to a rapid progression of DR. These risk factors encompass the age, pregnancy, duration of diabetes, body weight, control of blood glucose levels, retinal conditions, hypertension, hyperlipidemia, and renal diseases (17–29, 31, 32, 35–37, 39–43, 46–48, 50–53, 55–67, 69, 70, 72, 73, 85). The knowledge about DR varied among different categories of the HCPs involved in diabetic care, including those providing DR screening services. Most HCPs (93.8%) were aware that diabetes can cause eye damage, leading to irreversible vision impairment or loss.

Nine of the 48 studies reported that 100% of HCPs had a good knowledge of DR, and these studies were from Nigeria, South Africa, the UK, India, Thailand, Kenya, Saudi Arabia, Fiji, and China (65–67, 69, 73). A study conducted in Southern India among 200 paramedical personnel reported that only 2.5% of the 200 paramedical personnel knew about DR. Low proportion was attributed to insufficient educational materials on DR for this category of HCPs (63). In Northwestern Nigeria, 63.2% of the 105 physicians knew the effective method for prolonging the onset and progression of DR (17). In the Canary Islands, the results showed that 68.4% of the 165 sampled PHC nurses knew how to differentiate normal retinal images or photographs from the affected ones (42).

### 3.7 Attitudes of healthcare professionals toward diabetic retinopathy

Thirty-two studies that evaluated the attitudes of HCPs toward DR screening and referrals utilized common items in the Likert scale format (17, 18, 21–23, 28, 30, 39, 57, 65, 70–72). Some of these items reflected beliefs such as eye examinations are not within the realm of responsibility for the general healthcare providers or primary care physicians, eye-related problems are time-consuming, addressing diabetic retinopathy in an outpatient clinic setting is impractical, and performing fundoscopy without periodic in-service training is not valuable.

The proportion of HCPs with a positive attitude toward DR ranged from 13.2 to 100%. Notably, four studies conducted in Yemen, two in Indonesia, and Pakistan reported that 100% of all HCPs exhibited positive attitudes toward DR screening, including prevention (30, 39, 58, 69). These studies revealed that HCPs demonstrated commendable attitudes toward DR by prioritizing DR screening in diabetic patients. Additionally, HCPs in these studies received specialized training on DR, comprehended its psychological impact, respected the autonomy of diabetic patients, and emphasized the significance of stringent blood glucose control (30, 39, 58, 69). Conversely, a study in Saudi Arabia among 99 primary care physicians (PCPs), revealed that only 13.2% had positive attitudes toward DR (52). The study reported that PCPs believed that well-trained HCPs (the ophthalmologists, optometrists, and ophthalmic nurses) should conduct DR screening, including diagnosing and managing individuals affected by DR effectively, as opposed to the general PCPs, like GPs, family physicians, internists, and other non-ophthalmic practitioners involved in the management of diabetes.

### 3.8 Healthcare professionals’ practices of diabetic retinopathy

Forty studies used items such as screening for DR among all diabetes patients irrespective of the type, whether a patient was symptomatic, and adherence to standardized diabetic eye screening schedules and referral guidelines. Only 4.3% of Indonesia’s 92 general practitioners (GPs) had followed the DR referral and screening protocols effectively (58). This low proportion of GPs was mainly due to the lack of comprehensive vision testing, the unavailability of vision acuity testing charts and ophthalmic medical technology such as ophthalmoscopes for a basic fundoscopic examination, and an underdeveloped referral system (58). The practice of performing fundoscopy varied among PHC nurses and ophthalmic care practitioners (such as optometrists, ophthalmologists, and ophthalmic nurses) in eight studies conducted across five different regions, including Saudi Arabia, the USA, India, Australia, and South Africa (15, 16, 33, 44, 47, 54, 69, 71). Two South African studies conducted in the eThekwini municipality and the Waterburg and Capricorn Districts reported that all PHC nurses did not implement DR screening programs due to a lack of appropriate skills to perform screening procedures for DR, being busy with other responsibilities, staff shortage, and proactively referring them to ophthalmologists for eye examinations, regardless of visual symptoms (15, 16). In these two studies, 43% of 42 PHC nurses in eThekwini and all PHC nurses in the Waterburg and Capricorn districts only perform case history taking, including referring patients with diabetes to ophthalmic nurses (15, 16).

## 4 Discussion

This narrative review found variations in HCPs’ knowledge, attitudes, and practices regarding diabetic retinopathy (DR). While these disparities may have been due to the differences in tools, settings, and research methodologies, some commonalities were noted. Not unexpectedly, the educational background of HCPs plays a crucial role; those who received specialized training in ophthalmic care, such as ophthalmologists, optometrists, and ophthalmic nurses, typically scored higher across all three domains than other categories of HCPs in three WHO regions, namely Africa, the Americas, and South East Asia (15, 16, 44, 47, 55, 56, 69, 72). Primary care physicians (PCPs) and general practitioners (GPs) demonstrated a good knowledge of DR occurring due to prolonged hyperglycemia and when to screen patients diagnosed with diabetes in the Eastern Mediterranean, South East Asia, and European regions (22–24, 29, 50, 52, 62, 66, 70).

Positive attitudes of HCPs are important in the success of screening programs for DR. Healthcare professionals with a positive disposition toward DR screening were more likely to refer diabetic patients for DR screening (8, 28, 39, 45). A combination of adequate knowledge and appropriate training on screening for DR has been shown to positively influence attitudes (45, 69). It is crucial for all HCPs, regardless of their specialty, to be familiar with the global protocols for DR screening and understand that they have a responsibility to either screen or refer diabetic patients for eye-related issues.

As with knowledge, the practice of referring diabetic patients for DR screening varied among different categories of HCPs across all six regions. Poor practice was related to either insufficient resources or a lack of expertise. Non-ophthalmic trained nurses did not have the practical skills needed to perform basic eye examinations. Research has shown that training of non-ophthalmic HCPs in DR screening can be effective in improving early detection and appropriate referral of patients (89, 90). Foundational training for non-ophthalmic HCPs at the first point of care should be a priority area in all healthcare settings serving diabetic patients. This training should include fundamental skills such as visual acuity testing, ophthalmoscopy or fundoscopy, and being aware of DR referral guidelines.

The lack of resources such as ophthalmoscopes and dilating eye drops also contributed to poor practices in some settings, especially amongst general practitioners. Interestingly, this finding was not restricted to studies from low and middle-income regions, such as Africa and the Eastern Mediterranean region, but also reported in studies from Europe and America (17, 18, 22, 44, 53). Furthermore, some HCPs reported having too many other responsibilities that resulted in insufficient screening for DR in patients with diabetes (21, 37). These gaps result in many patients not being screened or referred for screening. Implementing educational measures is crucial for improving DR screening processes and developing an effective referral network to ophthalmologists or optometrists for comprehensive eye examinations for all diabetic patients.

The use of more sophisticated medical technology, such as artificial intelligence fundus imaging and optical coherence tomography, has enhanced screening for DR. While this review did not focus on how screening was done, it must be noted that the use of these technologies may enable earlier and more accurate detection and timely treatment. These technologies are powered by automated retinal image analysis, which is also suitable for non-dilated pupils for fundoscopy to save time during DR screening.

This review has some limitations. It was limited to non-experimental research on HCPs’ knowledge, attitudes, and practices toward DR screening and referrals. The literature search was confined to electronic data sources. The review exhibited susceptibility to publication bias, as studies that yielded statistically significant results were more likely to be published, potentially distorting the overall findings. The study design and sampling methodologies may have influenced the validity of the findings. The results of studies that used non-random sampling or had low response rates may not reflect the KAP of all HCPs in that setting. Furthermore, practice was self-reported, including non-random samples, and this is likely to differ from the actual KAP. The heterogeneity of studies and samples poses a challenge in synthesizing the findings of the review. Whilst these limitations significantly influence the conclusions drawn from the reviewed articles, this review still has value in identifying the gaps in existing KAP amongst HCPs across regions.

## 5 Conclusion

There are important gaps in the knowledge, attitudes, and practices regarding DR screening among HCPs, particularly non-ophthalmic-trained HCPs. These HCPs have limited knowledge of the risk factors, early signs, and progression stages of DR, and attitudes that reflect that DR screening should be the responsibility of ophthalmic-trained HCPs only. Screening for DR was poorly practiced. The common reasons for these gaps were inadequate training, insufficient screening resources, and a high workload. Regular in-service training is needed to enhance screening and timely referrals, particularly for non-ophthalmic professionals. It is imperative that, even at the level of primary healthcare, appropriate resources are available so that patients at risk of DR can be screened and referred appropriately to reduce the burden of visual impairment and blindness due to DR.
