# Supplementary material for: A narrative review of the knowledge, attitudes, and practices of healthcare professionals toward diabetic retinopathy
Source: Front Med (Lausanne). 2025 Aug 7;12:1536822. doi: 10.3389/fmed.2025.1536822 (PMC12367720; doi:10.3389/fmed.2025.1536822)
Supplement: Supplementary file 1 [file Table_1.docx]

**Supplementary Table 1:** JBI appraisal summary for analytic cross-sectional studies.

| **Studies** | **Types** | **Assessment items** | | | | | | | | **Total** | **Levels** | **Overall appraisal** |
| --- | --- | --- | --- | --- | --- | --- | --- | --- | --- | --- | --- | --- |
|  |  | **Q1** | **Q2** | **Q3** | **Q4** | **Q5** | **Q6** | **Q7** | **Q8** |  |  |  |
| Abdool et al. 2016 | Quantitative | ✓ | ✓ | ✓ | ✓ | ✓ | ✓ | ✓ | ✓ | 8/8 | Strong | Included |
| Abdool et al. 2020 | Quantitative | ✓ | ✓ | ✓ | ✓ |  |  | ✓ | ✓ | 6/8 | Moderate | Included |
| Abdulsalam et al. 2018 | Quantitative | ✓ | ✓ | ✓ | ✓ |  |  |  | ✓ | 5/8 | Moderate | Included |
| Abu-Amara et al. 2019 | Quantitative | ✓ | ✓ | ✓ | ✓ |  |  | ✓ | ✓ | 6/8 | Moderate | Included |
| Ahmed et al. 2020 | Quantitative | ✓ | ✓ | ✓ | ✓ |  |  | ✓ | ✓ | 6/8 | Moderate | Included |
| Al-Rasheed and Al-Adel 2017 | Quantitative | ✓ | ✓ | ✓ | ✓ |  |  | ✓ | ✓ | 6/8 | Moderate | Included |
| Alanazi et al. 2018 | Quantitative | ✓ | ✓ | ✓ | ✓ |  |  | ✓ | ✓ | 6/8 | Moderate | Included |
| Alasqah et al. 2020 | Quantitative | ✓ | ✓ | ✓ | ✓ |  |  | ✓ | ✓ | 6/8 | Moderate | Included |
| Al-Ghamdi et al. 2017 | Quantitative | ✓ | ✓ | ✓ | ✓ |  |  | ✓ | ✓ | 6/8 | Moderate | Included |
| Alhejji et al. 2020^30^ | Quantitative | ✓ | ✓ | ✓ | ✓ |  |  | ✓ | ✓ | 6/8 | Moderate | Included |
| Almoitairy et al. 2021 | Quantitative | ✓ | ✓ | ✓ | ✓ |  |  | ✓ | ✓ | 6/8 | Moderate | Included |
| Al-Rashidi et al. 2020 | Quantitative | ✓ | ✓ | ✓ | ✓ |  |  | ✓ | ✓ | 6/8 | Moderate | Included |
| Alsaedi et al. 2022 | Quantitative | ✓ | ✓ | ✓ | ✓ | ✓ | ✓ | ✓ | ✓ | 8/8 | Strong | Included |
| Alzaidi et al. 2016 | Quantitative | ✓ | ✓ | ✓ | ✓ |  |  | ✓ | ✓ | 6/8 | Moderate | Included |
| Anwar et al. 2019 | Quantitative | ✓ | ✓ | ✓ | ✓ | ✓ | ✓ | ✓ | ✓ | 8/8 | Strong | Included |
| Babelgaith et al. 2013 | Quantitative | ✓ | ✓ | ✓ | ✓ | ✓ | ✓ | ✓ | ✓ | 8/8 | Strong | Included |
| Babu et al 2021 | Quantitative | ✓ | ✓ | ✓ | ✓ |  |  | ✓ | ✓ | 6/8 | Moderate | Included |
| Bogunjoko 2015 | Quantitative | ✓ | ✓ | ✓ | ✓ |  |  | ✓ | ✓ | 6/8 | Moderate | Included |
| Barakat et al. 2023 | Quantitative | ✓ | ✓ | ✓ | ✓ | ✓ | ✓ | ✓ | ✓ | 8/8 | Strong | Included |
| Carlos et al. 2007 | Quantitative | ✓ | ✓ | ✓ | ✓ |  |  | ✓ | ✓ | 6/8 | Moderate | Included |
| Chelliah et al. 2020 | Quantitative | ✓ | ✓ | ✓ | ✓ |  |  | ✓ | ✓ | 6/8 | Moderate | Included |
| Daly 2014 | Quantitative | ✓ | ✓ | ✓ | ✓ | ✓ | ✓ | ✓ | ✓ | 8/8 | Strong | Included |
| Delorme 1998 | Quantitative | ✓ | ✓ | ✓ | ✓ |  |  | ✓ | ✓ | 6/8 | Moderate | Included |
| Dickson et al. 1996 | Quantitative | ✓ | ✓ | ✓ | ✓ |  |  | ✓ | ✓ | 6/8 | Moderate | Included |
| Edwiza et al. 2021 | Quantitative | ✓ | ✓ | ✓ | ✓ |  |  | ✓ | ✓ | 6/8 | Moderate | Included |
| Elnagieb and Saleem 2017 | Quantitative | ✓ | ✓ | ✓ | ✓ |  |  | ✓ | ✓ | 6/8 | Moderate | Included |
| Erdem 2020 | Quantitative | ✓ | ✓ | ✓ | ✓ |  |  | ✓ | ✓ | 6/8 | Moderate | Included |
| Fernꞻndez-Gutliѐrrez et al. 2023 | Quantitative | ✓ | ✓ | ✓ | ✓ | ✓ | ✓ | ✓ | ✓ | 8/8 | Strong | Included |
| Fatima and Ahmad 2018 | Quantitative | ✓ | ✓ | ✓ | ✓ |  |  | ✓ | ✓ | 6/8 | Moderate | Included |
| Foster 1996 | Quantitative | ✓ | ✓ | ✓ | ✓ | ✓ | ✓ | ✓ | ✓ | 8/8 | Strong | Included |
| George et al. 2019 | Quantitative | ✓ | ✓ | ✓ | ✓ |  |  | ✓ | ✓ | 6/8 | Moderate | Included |
| Gharsangi et al. 2021 | Quantitative | ✓ | ✓ | ✓ | ✓ |  |  | ✓ | ✓ | 6/8 | Moderate | Included |
| Ghosh 2007 | Quantitative | ✓ | ✓ | ✓ | ✓ |  |  | ✓ | ✓ | 6/8 | Moderate | Included |
| Goodman et al. 1997 | Quantitative | ✓ | ✓ | ✓ | ✓ | ✓ | ✓ | ✓ | ✓ | 8/8 | Strong | Included |
| El-Hajj et al. 2018 | Quantitative | ✓ | ✓ | ✓ | ✓ |  |  | ✓ | ✓ | 6/8 | Moderate | Included |
| Jagun et al. 2020 | Quantitative | ✓ | ✓ | ✓ | ✓ | ✓ | ✓ | ✓ | ✓ | 8/8 | Strong | Included |
| Khan et al. 2011 | Quantitative | ✓ | ✓ | ✓ | ✓ |  |  | ✓ | ✓ | 6/8 | Moderate | Included |
| Khandekar et al. 2008 | Quantitative | ✓ | ✓ | ✓ | ✓ |  |  | ✓ | ✓ | 6/8 | Moderate | Included |
| Kiely et al. 2017 | Quantitative | ✓ | ✓ | ✓ | ✓ |  |  | ✓ | ✓ | 6/8 | Moderate | Included |
| Kumar et al. 2020 | Quantitative | ✓ | ✓ | ✓ | ✓ | ✓ | ✓ | ✓ | ✓ | 8/8 | Strong | Included |
| Kupitz et al. 2014 | Quantitative | ✓ | ✓ | ✓ | ✓ | ✓ | ✓ | ✓ | ✓ | 8/8 | Strong | Included |
| Lestar et al. 2023 | Quantitative | ✓ | ✓ | ✓ | ✓ |  |  | ✓ | ✓ | 6/8 | Moderate | Included |
| Malik et al. 2023 | Quantitative | ✓ | ✓ | ✓ | ✓ | ✓ | ✓ | ✓ | ✓ | 8/8 | Strong | Included |
| McCarty et al. 2000 | Quantitative | ✓ | ✓ | ✓ | ✓ |  |  | ✓ | ✓ | 6/8 | Moderate | Included |
| Menash 2013 | Quantitative | ✓ | ✓ | ✓ | ✓ | ✓ | ✓ | ✓ | ✓ | 8/8 | Strong | Included |
| Muecke 2008 | Quantitative | ✓ | ✓ | ✓ | ✓ |  |  | ✓ | ✓ | 6/8 | Moderate | Included |
| Namperumalsamy et al. 2004 | Quantitative | ✓ | ✓ | ✓ | ✓ |  |  | ✓ | ✓ | 6/8 | Moderate | Included |
| Niyonsavye 2015 | Quantitative | ✓ | ✓ | ✓ | ✓ | ✓ | ✓ | ✓ | ✓ | 8/8 | Strong | Included |
| Oenga 2012 | Quantitative | ✓ | ✓ | ✓ | ✓ | ✓ | ✓ | ✓ | ✓ | 8/8 | Strong | Included |
| Pradhan et al. 2018 | Quantitative | ✓ | ✓ | ✓ | ✓ |  |  | ✓ | ✓ | 6/8 | Moderate | Included |
| Raman et al. 2006 | Quantitative | ✓ | ✓ | ✓ | ✓ |  |  | ✓ | ✓ | 6/8 | Moderate | Included |
| Shah et al 2017 | Quantitative | ✓ | ✓ | ✓ | ✓ | ✓ | ✓ | ✓ | ✓ | 8/8 | Strong | Included |
| Thirunavukkarasu et al. 2021 | Quantitative | ✓ | ✓ | ✓ | ✓ |  |  | ✓ | ✓ | 6/8 | Moderate | Included |
| Wright et al. 2001 | Quantitative | ✓ | ✓ | ✓ | ✓ |  |  | ✓ | ✓ | 6/8 | Moderate | Included |
| Xulu-Kasaba et al. 2021 | Quantitative | ✓ | ✓ | ✓ | ✓ | ✓ | ✓ | ✓ | ✓ | 8/8 | Strong | Included |

**Assessment questions (from the JBI checklist):**

**Q1**: “Were the criteria for inclusion in the sample clearly defined? **Q2**: Were the study subjects and the setting described in detail?” **Q3**: “Was the exposure measured in a valid and reliable way?” **Q4**: “Were objective, standard criteria used for measurement of the condition?” **Q5**: “Were confounding factors identified?” **Q6**: “Were strategies to deal with confounding factors stated?” **Q7**: “Were the outcomes measured in a valid and reliable way?” **Q8**: “Was appropriate statistical analysis used?”

**Responses**: “Yes, no, unclear, not applicable”

**Overall appraisal**: “Include, exclude, seek further info”

**Source**: <https://jbi.global/critical-appraisal-tools>
