# Supplementary material for: A narrative review of the knowledge, attitudes, and practices of healthcare professionals toward diabetic retinopathy
Source: Front Med (Lausanne). 2025 Aug 7;12:1536822. doi: 10.3389/fmed.2025.1536822 (PMC12367720; doi:10.3389/fmed.2025.1536822)
Supplement: Supplementary file 2 [file Table_2.docx]

**Supplementary Table 2:** JBI appraisal summary for qualitative studies.

| **Studies** | **Types** | **Assessment items** | | | | | | | | | | **Total** | **Levels** |
| --- | --- | --- | --- | --- | --- | --- | --- | --- | --- | --- | --- | --- | --- |
|  |  | **Q1** | **Q2** | **Q3** | **Q4** | **Q5** | **Q6** | **Q7** | **Q8** | **Q9** | **Q10** |  |  |
| Hipwell et al. 2014 | Qualitative | ✓ | ✓ | ✓ | ✓ | ✓ | ✓ |  | ✓ | ✓ | ✓ | 9/10 | Strong |
| Kumar et al. 2023 | Qualitative | ✓ | ✓ | ✓ | ✓ | ✓ |  |  | ✓ | ✓ | ✓ | 8/10 | Moderate |
| Ram et al. 2022 | Qualitative | ✓ | ✓ | ✓ | ✓ | ✓ |  |  | ✓ | ✓ | ✓ | 8/10 | Moderate |
| Yan 2012 | Qualitative |  | ✓ | ✓ | ✓ | ✓ |  |  | ✓ | ✓ | ✓ | 8/10 | Moderate |

**Assessment questions (from the JBI checklist):**

**Q1**: “Is there congruity between the stated philosophical perspective and the research methodology and the research methodology?” **Q2**: “Is there congruity between the research question or objective?” **Q3**: “Is there congruity between the research methodology and the methods used to collect data?” **Q4**: “Is there congruity between the research methodology and the representation and analysis of data?” **Q5**: “Is there congruity between the research methodology and the interpretation of results? **Q6**: “Is there a statement locating the researcher culturally or theoretically?” **Q7**: “Is the influence of the researcher on the research, and vice-versa, addressed?” **Q8**: “Are participants, and other voices, adequately represented?” **Q9**: “Is the research ethical according to current criteria or, for recent studies, and is there evidence of ethical approval by an appropriate body?” **Q10**: “Do the conclusions drawn in the research report flow from the analysis, or interpretation of the data?”

**Responses**: “Yes, no, unclear, not applicable”

**Overall appraisal**: “Include, exclude, seek further info”

**Source**: <https://jbi.global/critical-appraisal-tools>
